# Identification of Candidate Protein Biomarkers for CIN2+ Lesions from Self-Sampled, Dried Cervico–Vaginal Fluid Using LC-MS/MS

**DOI:** 10.3390/cancers13112592

**Published:** 2021-05-25

**Authors:** Ariadna Lara Gutiérrez, Julia Hedlund Lindberg, Ganna Shevchenko, Inger Gustavsson, Jonas Bergquist, Ulf Gyllensten, Stefan Enroth

**Affiliations:** 1 Department of Immunology, Genetics, and Pathology, Biomedical Center, SciLifeLab Uppsala, Uppsala University, SE-75108 Uppsala, Sweden; Ariadna.Laragutierrez.8612@student.uu.se (A.L.G.); julia.hedlund_lindberg@igp.uu.se (J.H.L.); inger.gustavsson@igp.uu.se (I.G.); ulf.gyllensten@igp.uu.se (U.G.); 2 Analytical Chemistry, Department of Chemistry-Biomedical Center, Uppsala University, SE-75237 Uppsala, Sweden; ganna.shevchenko@kemi.uu.se (G.S.); Jonas.Bergquist@kemi.uu.se (J.B.)

**Keywords:** cervical cancer pre-stages, cervico–vaginal fluid, FTA-cards, mass spectrometry, proteomics, biomarkers

## Abstract

**Simple Summary:**

Cervical cancer is the fourth most common cancer among women worldwide, and screening programs increase its detection rate and survivability. The molecular screening of the presence of human papilloma viruses (HPV) as alternatives to physical examinations offers cost-efficient solutions and can be performed on self-collected samples. A persistent infection with HPV is necessary (but not sufficient) to develop cancer, and additional biomarkers are needed to increase the precision. Here, we have analysed protein biomarkers found in self-collected dried cervico–vaginal fluid (CVF) from both controls and women with cervical cancer pre-stages. Our conclusion is that protein biomarkers can be robustly detected from dried CVF and that these can aid in the detection of cervical cancer pre-stages.

**Abstract:**

Molecular screening programs for cervical cancer detect the presence of human papilloma virus (HPV) in cell material or vaginal fluids. Persistent infection with high-risk HPV is a necessary pre-requisite, but the majority of infections do not lead to pathological states. Additional biomarkers are needed to increase the specificity of the molecular tests. Here, we have investigated the possibility of detecting protein biomarkers using mass spectrometry from dried self-sampled cervico–vaginal fluid deposited on FTA cards. We found significant intra-individual correlations (*p* < 2.2 × 10^−16^), although heterogenous protein profiles were obtained between individuals. Out of 3699 proteins found in total, 169 were detected in at least 95% of the samples. Using a discovery/replication design, 18 proteins were found to be significant in the discovery cohort, with higher values in those cases compared to controls. All of these were found to also have higher levels among the cases in the replication cohort, with one protein (DEAD-Box Helicase) remaining statistically significant. Finally, a predictive 7-protein multivariate model was developed with a sensitivity and specificity of 0.90 and 0.55, respectively. Our results demonstrate that robust measurements of protein biomarkers can be obtained from self-sampled dried CVF and that these could be used to predict cervical cancer pre-stages.

## 1. Introduction

Cervical cancer is currently the fourth most common worldwide cancer among women, with an incidence rate between 10.4 and 18.2 cases per 100,000 women, and reported mortality rates from 4.1 to 12.0 cases per 100,000 women [1]. In most cases, the cancer develops due to a persistent infection with oncogenic types of human papillomavirus (HPV). HPV infections are common and recurrent among women worldwide, and it has been estimated that around 85% of sexually active women will be infected at least once in their life with HPV. While low-risk HPV subtypes are associated with benign pathologies (such as genital warts), high-risk HPV subtypes are associated with malignant pathologies. Currently, 12 HPV types are considered high-risk for the development of cervical cancer. Although programmes for vaccination against high-risk HPV have been initiated for girls and boys in several countries, screening of the non-vaccinated population of sexually active women is essential to cancer detection [2]. A common screening technique is the Papanicolaou (also known as Pap-smear or cervical cytology) due to its simplicity, low cost, and possibility to detect pre-cancerous and cancerous lesions [3,4]. Cervical smears are examined via microscopy and classified according to the cytological conditions of the epithelial [5]. Pap-smear cytology, however, has a wide sensitivity window ranging from 55% to 94%, which can result in women with a cervical dysplasia not being identified [6]. An alternative method for the identification of women at risk of developing cervical cancer is testing for the presence of HPV. HPV tests either target the presence of high-risk HPV (hrHPV) DNA in the sample or expressed HPV genes in the form of RNA. These molecular tests have a higher sensitivity (but a lower specificity) than cytology. Recently, alternative screening approaches for cervical cancer have been suggested based on protein biomarkers [7]. We have previously identified a protein signature that can be used to identify cervical cancer based on plasma samples [8]. Although promising results were obtained, plasma does not allow for testing of HPV DNA or RNA, and proteins present in plasma are not necessarily detectable in cervico–vaginal fluid (CVF), for instance, which is being used for HPV DNA testing [9]. However, if complementary techniques could be employed using the same, minimally invasive sample type, it would be advantageous and beneficial for patients and clinicians [5,10]. 

In order to identify protein biomarkers that are unique to cervical cancer and that could be used simultaneously with HPV DNA testing, it would be necessary to study a biological fluid specific to the genital tract. Several studies have characterized the CVF proteome [11,12,13]. In particular, Van Raemdonck and colleagues [11] used mass spectrometry (MS) to identify 474 unique proteins in fresh frozen CVF from 12 (six healthy and six cervical cancers) samples, and Boylan and colleagues [13] described a “normal pap test core proteome” consisting of 153 proteins based on studies of up to 72 samples, also using MS. Using pooled CVF from 40 women, they also characterized a total of 714 proteins, illustrating the heterogeneity between individual CVF proteomes [13]. 

We have previously shown that the FTA elute filter paper cards is suitable for the self-sampling of cervico–vaginal fluid for HPV DNA testing [14]. Samples of dried CVF deposited on FTA cards is also amendable to analyses of protein biomarkers using ultra-sensitive high-throughput proteomics methods, like proximity extension assay (PEA) [15]. To the best of our knowledge, dried CVF deposited on FTA-cards has not been evaluated with MS-based proteomics or for large-scale investigations of protein biomarkers for cervical cancer pre-stages. The present study aims to investigate the use of dried CVF samples on FTA cards as a sample matrix for the discovery and validation of protein biomarkers in cervical cancer, characterized with MS-based proteomics. 

## 2. Materials and Methods

### 2.1. Sample Collection

The samples used have previously been described and used for HPV DNA testing and for the analysis of the vaginal microbiota [16,17]. The FTA elute micro cards were used in a large study investigating HPV-DNA testing based on home-sampled CVF. In brief, women participating in the study received a sample kit and a step-by-step instruction describing how to perform the vaginal fluid collection at home. This methodology have been previously described in Gustavsson et al. [18]. Briefly; (1) the woman has to insert the sampling brush (Viba-brush, Rover Medical Devices BV, Oss, The Netherlands) approximately 7 cm into the vagina, twist once, and remove; (2) the woman then has to place the sampling brush onto the FTA card, rotating one circle into the coloured area;(3) the woman then lets the card dry a few minutes, (4) lastly, she must fold the lid, place the card into the pre-addressed and pre-paid envelope, and send it to the laboratory by regular mail. Women that were positive for any of the 12 hrHPVs took a second HPV-test approximately 3 months after the first. The clinically validated HPVIR test [19] was used to examine HPV-positivity in the samples. If the second test was also positive, the women were referred to further investigations as defined in the Swedish Cervical Cancer Screening programme. Here, samples from HPV-negative women and the second sample from double positive women that were later diagnosed through histology with CIN2+ were used. The samples were collected from pre-menopause women, but not during menstrual bleeding. 

### 2.2. Mass Spectrometry Analysis and Quantification

#### 2.2.1. Sample Preparation

The MS-analyses, including a bioinformatic analysis of the raw data, were performed by the Mass Spectrometry facility at the Biomedical Centre, Uppsala, Sweden. In brief, FTA cards were punched, and one (replication cohort) or two (discovery cohort) 3.5 mm discs per patient sample was used. The proteins were processed with a previously described on-filter digestion protocol [20] using 3 kDa centrifugal filters (Millipore). The samples were reduced and alkylated in 1.5 mL protein low bind Eppendorf tubes prior to the on-filter desalting and digestion. Centrifugation was carried out using a centrifugal force of 14,000× *g* throughout the protocol. A volume of 10 µL of 4 5 mM aqueous DTT was added to all samples, and the mixtures were incubated for 15 min at 50 C to reduce the disulfide bridges. The samples were cooled down to RT, 10 µL of 100 mM aqueous IAA was added, and the mixtures were incubated for an additional 15 min at RT in darkness to carabamidomethylate the cysteines. The samples were transferred to spin filters that had been pre-washed with 250 µL of 20% ACN for 15 min and then 500 µL of MQ water for 20 min. The samples were then centrifuged for 10 min to remove the added salts, detergents, and other interfering substances. An additional volume of 150 µL of 20% ACN in 50 mM NH_4_HCO_3_ was added and the filters were spun for 10 min followed by 200 µL of 50 mM NH_4_HCO_3_, and centrifugation for another 10 min. Finally, a volume of 100 µL of 50 mM NH_4_HCO_3_ was added together with trypsin to yield a final trypsin/protein concentration of 5% (*w*/*w*). The tryptic digestion was performed at 37 °C in darkness. The samples were then centrifuged for 20 min to collect the tryptic peptides. An additional volume of 100 µL of 20% ACN, 1% HAc was added and the filters were spun for 10 min and pooled with the first tryptic peptide filtrate. The collected peptide filtrate was then vacuum centrifugated to dryness using a Speedvac system ISS110 (ThermoFisher Scientific, Massachusetts, USA). The dried peptides were resolved in 60 µL of 0.1% formic acid and further diluted five times prior to nano-LC-MS/MS. The resulted peptides were separated in reverse phase on a C18- column with 90 min gradient and electro-sprayed on-line to a Q-Exactive Plus mass spectrometer (Thermo Finnigan, Bremen, Germany). Tandem mass spectrometry was performed, applying higher-energy collisional dissociation. 

#### 2.2.2. LC-MS/MS

The samples were analysed using a Q Exactive Plus Orbitrap mass spectrometer (Thermo Fisher Scientific, Bremen, Germany) equipped with a nano-electrospray ion source. The peptides were separated via reversed-phase LC using an EASY-nLC 1000 system (Thermo Fisher Scientific). A set-up of a pre-column and an analytical column was used. The pre-column was a 2-cm EASY-Column (ID 100 µm, 5 µm C18; Thermo Fisher Scientific) and the analytical column was a 10-cm EASY-Column (ID 75 µm, 3 µm, C18; Thermo Fisher Scientific). Peptides were eluted with a 90 min linear gradient from 4% to 100% acetonitrile at 250 nL min^−1^. The mass spectrometer was operated in positive ion mode, acquiring a survey mass spectrum with resolving power 70,000 (full width half maximum), *m*/*z* = 400–1750, using an automatic gain control target of 3 × 10^6^. The 10 most intense ions were selected for higher-energy collisional dissociation fragmentation (25% normalized collision energy) and MS/MS spectra were generated with an automatic gain control target of 5 × 10^5^ at a resolution of 17,500. The mass spectrometer operated in data-dependent mode.

#### 2.2.3. MS Data Handling

The acquired data (RAW files) were processed using MaxQuant software, version 1.5.1.2, and database searches were performed using the implemented Andromeda search engine [21]. MS/MS spectra were correlated to a FASTA database containing proteins from *Homo sapiens* extracted from the UniProt database (release date: September 2018). A decoy search database, including common contaminants and a reverse database, was used to estimate the identification false discovery rate where a rate of 1% was accepted. The search parameters included: maximum 10 ppm and 0.6 Da error tolerances for the survey scan and MS/MS analysis, respectively; enzyme specificity was trypsin; maximum one missed cleavage site allowed; cysteine carbamidomethylation was set as static modification; and oxidation of methionine was set as variable modification. The search criteria for protein identification were set to at least two matching peptides of 95% confidence level per protein.

### 2.3. Statistical Analysis

All calculations and illustrations were carried out in R Studio [22] (version 3.6.2). Raw protein data abundance levels (no prior normalization) from MaxQuant were first normalized on an individual level. For the duplicated samples in the replication cohort, the mean values between the replicates were used. The normalization was conducted by dividing the values for each protein by the sum of all protein values for that individual (Equation (1)).

(1) $$Normalized\;protein\;abundance=\frac{data\;\left[individual\;\times \;protein\right]}{sum\left(data\left[individual\;\times \;protein\right]\right)}$$

Statistical significance for continuous measurement was evaluated using the two-sided Wilcoxon ranked sum test and with the Fisher’s exact test for the binomial analyses (above or below detection limit). The continuous data was z-scaled to normality before building the multivariate models. This was done separately in the discovery and replication cohorts for each protein, subtracting the mean (after removing observations below detection limit) and dividing with the standard deviation (after removing observations below detection limit). The feature selection for the multivariate analyses was carried out using recursive feature elimination as implemented by the ‘rfe’-functions in the ‘caret’ R-package [23]. In the ‘caret’ package [23] implementation, the performance of a defined subset size of ingoing variables was compared to the performance using all variables, and the best performing size was selected. Here, sizes of 3 to 15 were specified. After feature selection, naïve Bayes models were built with the ‘caret’ R-package using a cross-validation schema to optimise the parameters. Half (50%) of the samples were used to train and test the models and the remaining 50% to finally evaluate the performance. Plotting of receiver operating characteristics (ROC) curves, calculations of AUCs (area under curve), point estimates of sensitivity and specificity, and testing of difference in AUCs (DeLong’s method) were conducted using the pROC package [24]. Beanplots were generated using the ‘beanplot’-package [25]. 

## 3. Results

### 3.1. Dried CVF Is Suitable for Proteomic Analyses with Mass Spectrometry

A total of 103 samples from 99 FTA cards with dried self-sampled CVF were analysed with MS (Table 1). These samples were collected previously in a randomized controlled trial comparing self-sampled CVF with conventional Pap-smear cytology in cervical cancer screening [16]. Women were selected as controls for the present study if they had normal cytology and negative HPV DNA test results for 12 high-risk HPVs (16, 18, 31, 33, 35, 39, 45, 51, 52, 56, 58, and 59) using the clinically validated HPVIR test [19]. Women defined as cases had persistent HPV infection, defined as having two HPV positive tests approximately 3 months apart and a cervical histology with cervical intraepithelial neoplasia (CIN) of grade 2 or higher. The FTA samples were divided into a discovery and replication cohort (Table 1) and analysed with MS (Methods) in two separate batches. Overall, we detected a total of 3697 different proteins in at least one individual (Table S1). In any of the subgroups, the number of detected proteins ranged from 1654 to 2724. Four (4) samples from the replication cohort were analysed in duplicates (Figure S1) and the results showed significant correlations (*p* < below machine precision, 2.2 × 10^−16^, two-sided Spearman’s text), with correlation coefficients ranging from 0.79 to 0.85. 

**Figure 1:**
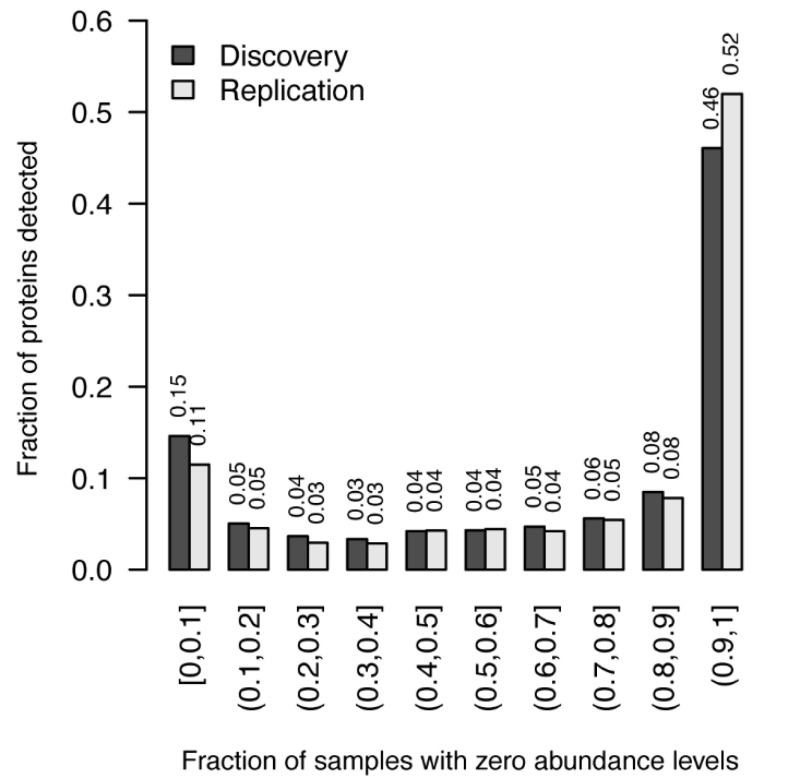
Protein detection rates. The fraction of protein (y-axis) with a specific zero-detection rate across samples (x-axis) for the discovery and age-matched replication cohorts (Case1 and Control in Table 1). For instance, 46 and 52% of the proteins detected in at least one sample were not detected in 90% or more of all analysed samples, while 15 and 11% of the proteins were detected in at least 90% of the samples.

Focusing on proteins that were detected in at least 95% of the individuals within subgroups, we identified between 247 and 494 proteins in the different subgroups, with 169 proteins being in common to all subgroups. We compared the set of detected proteins here with the set of 153 proteins defined as the Normal Pap Test Core Proteome (NPTCP) in Boylan [13]. Boylan and colleagues defined this set as proteins detected in at least four of the five samples of residual pap-smear fluid from healthy women. Of the 153 proteins in the NPTCP, we found 96.7 to 97.4% in at least one of our control groups. When restricting the comparison to proteins found in at least 95% of the individuals in the control groups, we detected 53.6 to 66.0% of the proteins in the NPTCP. This very high variability between the CVF proteome of individual women is illustrated in Figure 1, where the non-detection fraction in samples is shown for both the discovery and the age-matched replication cohorts. Close to 50% of the proteins (46% and 52% respectively) in both cohorts were not detected in at least 90% of the samples (Figure 1).

### 3.2. Univariate Protein Biomarkers for CIN2+

**Figure 2:**
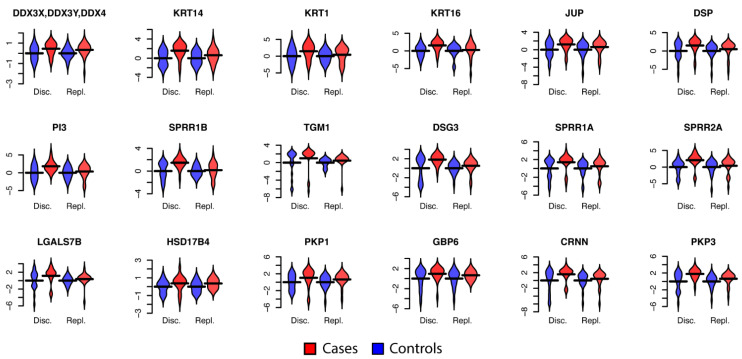
Distribution of protein abundance in women with CIN2+ (Cases) and women with HPV-negative samples (Controls). Protein abundance levels (log2) in both discovery and replication cohorts for the 18 proteins with a nominally significant *p*-value (*p* < 0.05, Wilcoxon test) in the discovery cohort. Protein abundance levels are shifted by the mean of the controls in the discovery and replication cohorts, respectively. This shifts the mean of the controls to 0 with the increased levels in cases easily visible.

**Figure 3:**
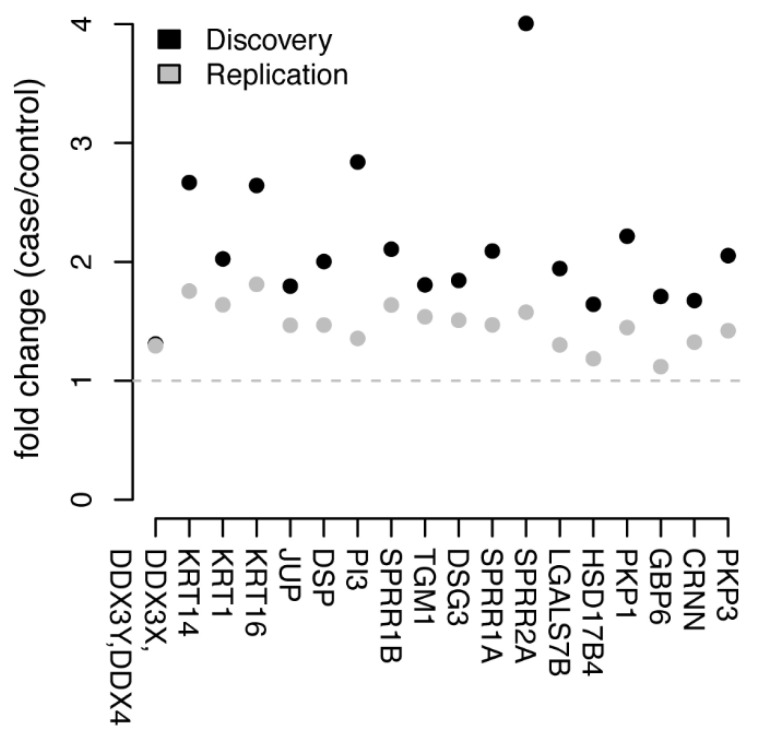
Trend of protein abundance as fold-change. Comparison between the discovery (black) and the replication (grey) cohorts of the trend distribution among CIN2+ and HPV-negative samples. Ratio values represented as fold-change, where values above one (1) represent CIN2+ with higher protein abundance than HPV-negative samples.

The proteomics data was first normalized by dividing each sample’s individual protein abundance counts with the sum of all counts for that individual (Methods). This ensures that the abundance profiles are on a comparable scale between individuals and across cohorts. Requiring a protein-detection rate of at least 75% of the samples and higher mean abundance levels in cases compared to controls resulted in 207 proteins from the discovery cohort that were kept for further analysis (Table S1). In total, 18 (CRNN, DDX3X/DDX3Y/DDX4, DESP, DHB4, DSG3, ELAF, GBP6, K1C14, K1C16, K2C1, LEG7, PKP1, PKP3, PLAK, SPR1A, SPR1B, SPR2A, and TGM1, Figure 2) of these 207 proteins were found to have nominally significant (*p* < 0.05, Wilcoxon ranked sum test) differences in abundance levels between cases and controls. Among the 207 proteins, 168 (81.2%) were present in at least 75% of the samples in the replication cohort, including the 18 selected proteins from the discovery data. We then examined these 18 proteins in the replication cohort, and all 18 had higher abundance levels in cases compared to control (Table S1, Figure 2 and Figure 3). However, only one of the proteins (DDX3X/DDX3Y/DDX4) was found to also shave nominally significant differences in abundance levels (*p* = 0.025, Wilcoxon ranked sum test, Table S1) in the replication cohort. 

In addition to the analysis of commonly present proteins, we also investigated the protein detection fraction between cases and controls. In the discovery data, a total of 3225 proteins were detected in at least one sample. For each of these, the number of samples with detected abundance levels in cases was compared to the number in controls using a two-sided Fisher’s exact test. For 97 of the proteins, we found nominally significant (*p* < 0.05) differences in protein detection fraction between cases and controls. Nine (9) of these were found to have higher detection frequencies in cases than in controls. 90 of these 97 proteins (92.7%) were also detected in the replication cohort, including eight of the proteins with higher detection fraction in cases in the discovery data. Seven of these eight proteins (87.5%) were also found to have higher detection fraction in the replication data, and 55 of 82 proteins (67.1%) with a lower detection rate in discovery were also lower in the replication data. Four (4) of the 90 proteins were also found to have nominally significant differences in detection rate in the replication data. For all four proteins, the detection fractions differences had the same direction in the discovery and replication data. The proteins, *p*-values, and detection rates for both the discovery and replication data are summarized in Table S1. 

### 3.3. Multivariate Biomarker Signature for CIN2+

**Figure 4:**
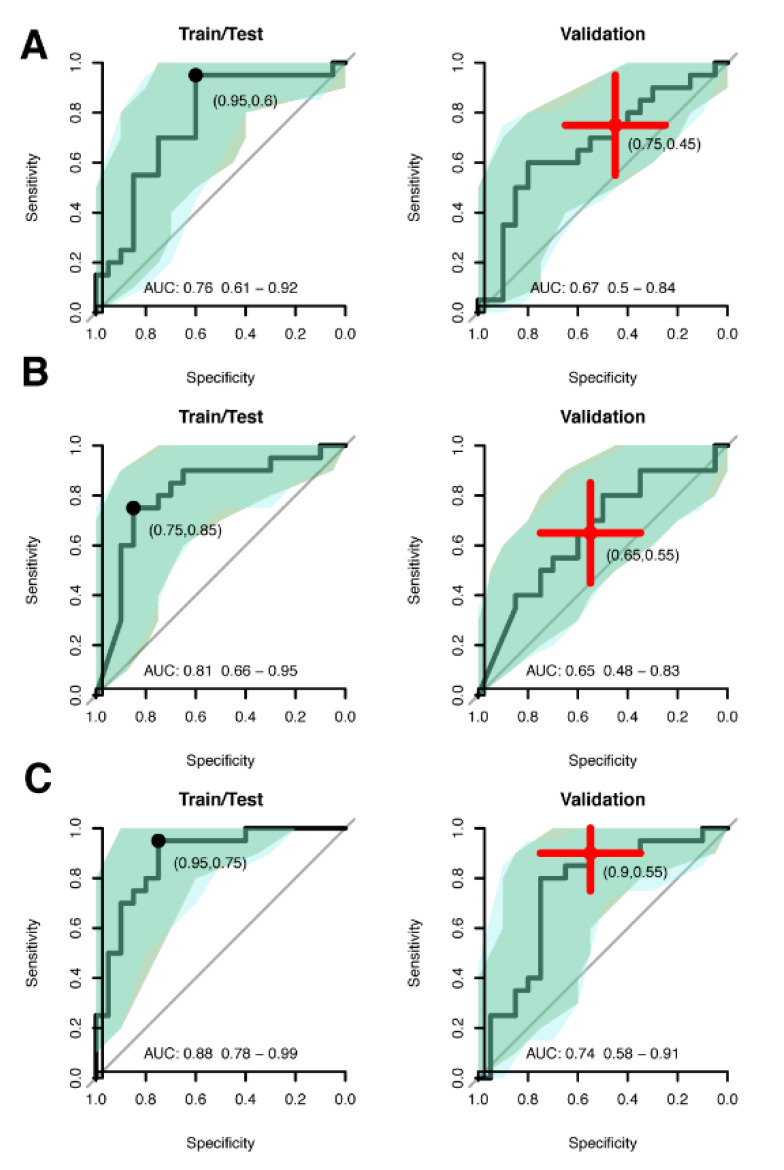
Multivariate analysis performance. (**A**) ROC curve for the training and test proportion of the data (left, 50% of the samples) and for the validation proportion of the data (50% of the samples). The model is based on protein with a continuous distribution of abundance only. The marked point in the left panel curve corresponds to a cut-off representing the best point (closest to perfect classification), and the point estimate of sensitivity and specificity for that cut-off is written out. The red cross on the right panel corresponds to the 95% confidence interval of sensitivity and specificity at the cut-off defined in the training/test set but in the validation set. (**B**) As A, but for the proteins with binomial (i.e., above or below LOD) representation. (**C**) As A, but for a model based on both the proteins with a continuous abundance and those with a binomial representation.

The common available proteins (168 and 90) from the univariate analysis were included in the multivariate analysis. The 168 proteins with continuous abundance levels were first normalized (z-score) in each of the two cohorts separately to ensure comparable levels. Given that the sample size here is limited, we created a combined data set with all 80 samples from both cohorts. Firstly, the 168 proteins and individual age were used, and the data was split into training proportion (50%) and a validation proportion (50%). The training proportion was first used to carry out feature selection (Methods), which returned six proteins (Ras-related protein Rab-6A (UniprotID: P20340), Ubiquitin-40S ribosomal protein S27a (P62979), Tripartite motif-containing protein 29 (Q14134), Zinc finger protein 185 (O15231), Keratin, type I cytoskeletal 14 (P02533), Envoplakin (Q92817)). A naïve Bayes discriminator was optimised using a 3-fold cross-validation schema with the six selected proteins. The performance of the model was then estimated using a mean across the cross-validation splits (Figure 4A, left panel) and in the validation proportion of the data (Figure 4A, right panel). A specific cut-off for calling cancer was taken at the point closest to perfect classification (Methods). The generated model achieved an AUC of 0.67 in the validation proportion of the data, which was not statistically different (Table 2) from that obtained during training and testing (AUC = 0.76). The point-estimates of sensitivity (0.75) and specificity (0.45) in the validation proportion at the cut-off generated in the training fell within the 95% confidence intervals of the estimates during training (Table 2). 

**Figure 5:**
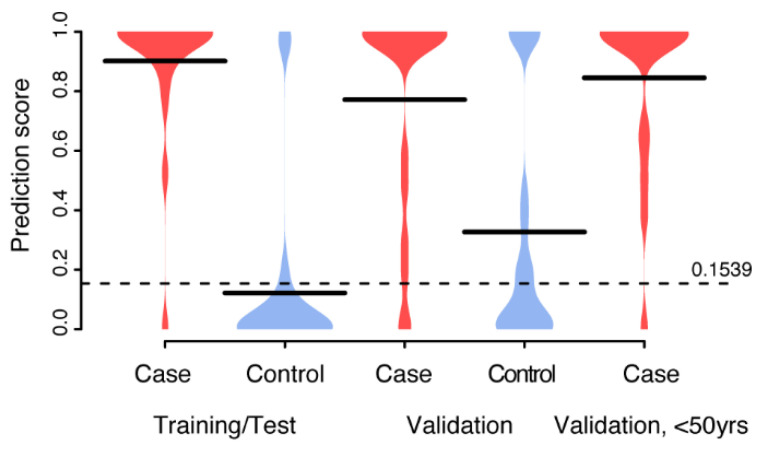
Distribution of prediction scores from the combined model. Prediction scores across the training/test, validation and the third replication data (Case 2 in Table 1) datasets. The dotted horizontal line corresponds to the cut-off (0.1539) defined during training. Thick black lines indicate mean values.

Secondly, we repeated the analyses as above but using the 90 proteins with binominal representation and individual age (Figure 4B). Here the feature selection returned all proteins and age and a model was built in the same way as above. Again, the performance estimates in the training and validation proportion of the data did not differ significantly and was similar to that obtained with the six proteins with the continuous measures used above (Table 2). Lastly, we used a combination of the 168 plus 90 proteins and individual age and repeated the analyses as above (Figure 4C). The returned model consisted of seven proteins, out of which three (Ras-related protein Rab-6A (UniprotID: P20340), Ubiquitin-40S ribosomal protein S27a (P62979), and Tripartite motif-containing protein 29 (Q14134)) came from the continuous data and four (Prohibitin-2 (Q99623), Actin-related protein 2/3 complex subunit 5 (O15511), Lysine-tRNA ligase (Q15046), and Testin (Q9UGI8)) came from the binomial. This combined model had an AUC of 0.74 and achieved a sensitivity and specificity of 0.90 and 0.55, respectively, in the validation proportion of the data (Table 2). Lastly, the combined model was applied to the second case group (Case 2, Table 1) from the replication data. This set of women is significantly younger (*p*-values < 9.4 × 10^−8^, Wilcoxon ranked sum test) compared to other cohorts (Table 1) and were only positive for HPV16, while the other cases have a mixed HPV profile (Table 1). The distribution of prediction scores from this group (Figure 5) were not significantly different from the other cases (all *p*-values < 0.25, Wilcoxon ranked sum test), but were significantly higher than for the control cohorts (all *p*-values < 5.48 × 10^−4^, Wilcoxon ranked sum test). 

## 4. Discussion

At-home self-sampling of CVF for the purpose of screening for HPV in relation to cervical cancer is cost-efficient and reliable [9]. We have previously conducted a randomized study using the FTA card for at-home self-sampling of CVF for screening of HPV, and shown that up to two times as many cancer pre-stages can be detected compared to using conventional cytology [16]. The majority of HPV infections are transient and does not lead to lesions or invasive cervical cancer, and additional biomarkers would be useful for predictions of clinical outcome for the women identified as HPV infected in screening. We have previously shown that cervical cancer cases can be separated from healthy controls based on the analysis of circulating protein biomarkers in plasma at the time of diagnosis [8]. However, plasma is not a suitable sample matrix for at-home sampling and is not an optimal sample for the detection of HPV. Analysis of protein biomarkers in plasma together with HPV testing based on CVF would require a visit by the woman to a healthcare centre for sampling, which eliminates the advantages of at-home self-sampling. Previous studies have shown that liquid residual pap-smear fluid can be used for proteomics analysis [13] and we have shown that the FTA card can be used for the detection of proteins using the affinity-based protein extension assay (PEA) [15,26]. Here, we have used MS analysis of the samples collected on FTA cards from our previous randomized study, to compare the CVF proteome in women with histology defined CIN2+ at diagnosis and an on-going persistent HPV infection with HPV-negative women. 

Our results show that self-sampled dried CVF deposited on the FTA card is amendable to proteomics analysis using MS. The results further demonstrate that the individual CVF proteome profile is highly diverse, corroborating the conclusions of previous studies [11,13]. Interestingly, the CVF include a very large number of proteins, many at low abundance and with strong individual variation, posing a real challenge for any detection method. This also means that it is difficult to pinpoint a set of proteins that are common to all women. We therefore restricted the analyses to proteins that were either detected in significantly different fractions (regardless of concentration) between cases and controls or with continuous measurements detected in at least 95% of the samples and had higher levels in cases compared to controls. Using multivariate machine learning, we identified a signature of seven (7) proteins that were able to separate cases from controls with a sensitivity of 0.95 and a specificity of 0.75. A similar performance (i.e., not statistically significantly different) was then obtained in the validation proportion of the data. 

Three of the proteins in the seven-protein model came from the analysis of continuous measurements and four proteins from the analysis of different fractions in cases compared to controls. The four proteins with different fractions were Prohibitin-2 (UniprotID: Q99623, coding gene: PHB2), Actin-related protein 2/3 complex subunit 5 (O15511, ARPC5), Lysine-tRNA ligase (Q15046, KARS1) and Testin (Q9UGI8, TES). According to the Human Proteome Atlas (HPA), all of these proteins are expressed in many tissue types, including female reproductive tissue. None of these proteins have been associated with cervical cancer in the literature, but the Human Proteome Atlas (HPA, http://www.proteinatlas.org accessed on 11 January 2021) [27] lists Prohibitin-2 as an unfavourable biomarker for endometrial cancer, Actin-related protein 2/3 complex subunit 5 as an unfavourable biomarker for both renal and liver cancer, Lysine-tRNA ligase as an unfavourable biomarker for head and neck cancer, and, finally, Testin as favourable biomarker for renal cancer and an unfavourable biomarker for pancreatic cancer. The three proteins with higher concentrations in cases compared to controls included in our model were; Ras-related protein Rab-6A (P20340, RAB6A), Ubiquitin-40S ribosomal protein S27a (P62979, RPS27A) and Tripartite motif-containing protein 29 (Q14134, TRIM29). Again, the HPA indicates that these are ubiquitously expressed across tissues, including the female reproductive tissue. Ras-related protein Rab-6A was also listed as an unfavourable biomarker for endometrial, liver and pancreatic cancers, Ubiquitin-40S ribosomal protein S27a as unfavourable biomarker for renal and liver cancer, and Tripartite motif-containing protein 29 as an unfavourable biomarker for endometrial, stomach and pancreatic cancer. Previous studies have reported Ubiquitin-40S ribosomal protein S27a as being present in higher amounts in the urine of cervical cancer patients compared to controls when measured with mass spectrometry [28] and an overexpression of Tripartite motif-containing protein 29 (both mRNA and protein) in cervical cancer tissue compared to matched adjacent cervical tissues [29]. 

The strengths of this study include using age-matched sets of cases and controls with high-quality clinical data, including histology based on biopsy and testing for 12 high-risk HPV-types using a clinically validated HPV-test [19]. Furthermore, five of the samples were characterized with MS in duplicates (separate punches from the same FTA card), providing highly significant correlations between replicates (*p* < below machine precision, 2.2 × 10^−16^). Interestingly, even comparing the two sets of multiple punches from the same FTA card did not result in a 100% identical list of proteins detected. This strengthens the observation made previously by Boylan and colleagues [13] to define the Normal Pap Test Core Proteome (NPTCP) based on presence of a protein in four out of five individual proteomes. Here, the overall overlap between duplicate punches from the same individual was between 69.9% and 89.9% (comparing overlapping proteins to proteins detected in either replicate), suggesting that even higher observation rates could be needed to reach a certainty of which proteins that constitute a common set. Although a single punch is technically sufficient for analysis with MS, strategies based on analysing multiple punches from each sample could be an attractive solution for future studies, in order to account for possible intra-sample variance in protein detection rates as seen in the replicates here. 

The relatively small number of samples limits the current study. Even though all proteins with univariate significance in the discovery cohort has the same direction of change in the replication cohort, these changes were not large enough to reach statistical significance save for one protein. A second limitation is that the detected candidate protein biomarkers were not validated by a secondary technology with read-out in absolute concentrations, which could have helped in providing clinically relevant cut-offs. Finally, since we observed increasing abundance levels in cases as compared to controls, follow-up samples after treatment in the cases would have been needed to investigate if the abundance levels would return to normal. 

## 5. Conclusions

In conclusion, self-sampling of CVF onto the FTA elute micro card is suitable for proteomics analyses using mass spectrometry and several candidate protein biomarkers for prediction of CIN2+ lesions were identified. Our results however indicate that there is a large individual variance in the CVF proteome and further studies are needed to replicate the results and to evaluate their usefulness in screening.

## Figures and Tables

**Table 1:** Sample characteristics.

Cohort	N	Age ^a^	HPV-Status ^b^	No of Proteins ^c^	NPTCP % Overlap ^d^
**Discovery**					
Case	20	54.2 (50–60)	16 (45%), 33/52/58 (35%), 31 (15%), 39 (10%), 51 (10%), 18/45 (5%), 59 (5%)	2411 (360)	97.4 (56.2)
Control	20	53.8 (50–59)	Negative	2724 (494)	96.7 (66.0)
**Replication**					
Case1	20	50.8 (47–60)	16 (35%), 33/52/58 (35%), 51 (15%), 39 (10%), 56 (10%), 18/45 (5%), 31 (5%), 59 (5%)	2137 (332)	96.1 (53.6)
Case2	19	35.6 (30–45)	16 (100%)	1630 (247)	96.1 (43.1)
Control	20	50.9 (47–59)	Negative	2322 (329)	97.4 (53.6)
				3697 (169)	98.7 (37.9)

^a^ Mean and range. ^b^ Reported as HPV-type (% of samples with that type). Note that some samples were infected with multiple HPV-types and the sum could be over 100%. ^c^ Number of proteins detected in at least one sample, with number of proteins detected in at least 95% of the individuals in a cohort within parentheses. ^d^ Percentage of proteins in the Normal Pap Test Core Proteome (NPTCP, as defined in Boylan et al. [13]) found among the proteins in at least one individual and in the set of proteins detected in at least 95% of the individuals in a cohort within parentheses. Boldface Cohort subheading refers to samples used in the discovery and replication respectively.

**Table 2:** Performance estimates in the multivariate modelling.

	Model Size	AUC ^a^	AUC Difference ^b^	Sensitivity ^c^	Specificity ^d^
**Continuous**					
Train	6 proteins	0.76 (0.61–0.92)		0.95 (0.85–1.00)	0.60 (0.40–0.80)
Validation		0.67 (0.50–0.84)	0.44	0.75 (0.55–0.95)	0.45 (0.25–0.65)
**Binomial**					
Train	90 proteins + age	0.81 (0.66–0.95)		0.75 (0.55–0.95)	0.85 (0.70–1.00)
Validation		0.65 (0.48–0.83)	0.19	0.65 (0.45–0.85)	0.55 (0.35–0.75)
**Combined**					
Train	7 proteins	0.88 (0.78–0.99)		0.95 (0.85–1.00)	0.75 (0.55–0.90)
Validation		0.74 (0.58–0.91)	0.17	0.90 (0.75–1.00)	0.55 (0.35–0.75)

^a^ Point estimates of the AUC and 95% confidence intervals. ^b^ *p*-value denoting two-sided difference in the AUC point estimate between the train and test proportion, as calculated by the DeLong-method. ^c,d^ Point estimates and 95% confidence intervals. Bold font subheadings correspond to the three variants of multivariate models.

## Data Availability

The mass spectrometry proteomics data have been deposited to the ProteomeXchange Consortium via the PRIDE [30] partner repository with the dataset identifier PXD026064.

## Supplementary Materials

The following are available online at https://www.mdpi.com/article/10.3390/cancers13112592/s1, Figure S1: Distribution of detected proteins between replicates. Table S1: Detected proteins and univariate statistics.
