# Supplementary material for: Identification of Candidate Protein Biomarkers for CIN2+ Lesions from Self-Sampled, Dried Cervico–Vaginal Fluid Using LC-MS/MS
Source: Cancers (Basel). 2021 May 25;13(11):2592. doi: 10.3390/cancers13112592 (PMC8198222; doi:10.3390/cancers13112592)
Supplement: Supplementary file 1 [file cancers-13-02592-s001.zip › ALG_supplFigure_S1.pdf]

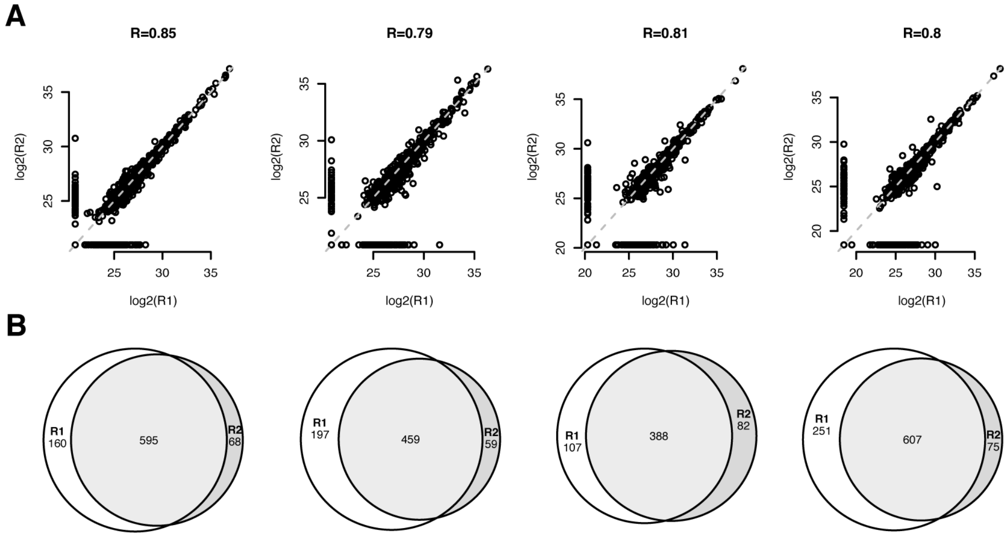

Supplementary Figure 1. Distribution of detected proteins between replicates. (A) Log2 values of the raw proteins abundance values in the two replicates are shown. Proteins under detection limits in either of the replicates are plotted out with artificial low values along each of the axes. **(B)** Proportional Venn diagrams illustrating the number of proteins above detection limit in each replicate and the overlap between these.
